# Cardiac troponin I and T for ruling out coronary artery disease in suspected chronic coronary syndrome

**DOI:** 10.1038/s41598-022-04850-7

**Published:** 2022-01-18

**Authors:** Sjur H. Tveit, Peder L. Myhre, Tove Aminda Hanssen, Signe Helene Forsdahl, Amjid Iqbal, Torbjørn Omland, Henrik Schirmer

**Affiliations:** 1 Department of Cardiology, Division of Medicine, Akershus University Hospital, Lørenskog, Norway; 2 Institute of Clinical Medicine, Faculty of Medicine, University of Oslo, Oslo, Norway; 3 Department of Health and Care Science, UiT - The Arctic University of Norway, Tromsø, Norway; 4 Department of Radiology, University Hospital of North Norway, Tromsø, Norway; 5 Department of Cardiology, University Hospital of North Norway, Tromsø, Norway

**Keywords:** Diagnostic markers, Cardiovascular diseases, Proteins

## Abstract

To compare the performance of high-sensitivity cardiac troponin I and T (hs-cTnI; hs-cTnT) in diagnosing obstructive coronary artery disease (CAD_50_) in patients with suspected chronic coronary syndrome (CCS). A total of 706 patients with suspected CCS, referred for Coronary Computed Tomography Angiography, were included. cTn concentrations were measured using the Singulex hs-cTnI (limit of detection [LoD] 0.08 ng/L) and Roche hs-cTnT (LoD 3 ng/L) assays. Obstructive coronary artery disease (CAD_50_) was defined as ≥ 50% coronary stenosis. Cardiovascular risk was determined by the NORRISK2-score. Median age of the patients was 65 (range 28–87) years, 35% were women. All patients had hs-cTnI concentrations above the LoD (median 1.9 [Q1-3 1.2–3.6] ng/L), 72% had hs-cTnT above the LoD (median 5 [Q1-3 2–11] ng/L). There was a graded relationship between hs-cTn concentrations and coronary artery calcium. Only hs-cTnI remained associated with CAD_50_ in adjusted analyses (OR 1.20 95% Confidence Interval [1.05–1.38]), *p* = 0.009). The C-statistics for hs-cTnI and hs-cTnT were 0.65 (95% CI [0.60–0.69]) and 0.60 (0.56–0.64). The highest specificity and negative predictive values for CAD_50_ were in the lowest NORRISK2-tertile. hs-cTn concentrations provide diagnostic information in patients with suspected CCS, with superior performance of hs-cTnI compared to hs-cTnT in regard to CAD_50_. The diagnostic performance appeared best in those with low cardiovascular risk.

## Introduction

Cardiac troponins (cTn) exist as two specific isotypes; I and T (cTnI, cTnT). Elevated concentrations of cTn are seen in a range of acute and chronic cardiac disease states, such as acute myocardial infarction (AMI), cardiac arrhythmias and heart failure^1^, as well as in non-cardiac disease^2^. In addition, cTn has been shown to be a robust marker of cardiovascular- and all-cause mortality, both in the general population^3–5^, in patients with known coronary artery disease (CAD)^6^, in patients with acute coronary syndrome (ACS)^7,8^, as well as in patients with non-cardiac disease^9^.

Currently, high sensitivity (hs) assays exist for both troponin isotypes, enabling accurate quantification of cTn even in patients without known cardiovascular disease (CVD)^10^. In addition to the widely adopted Roche hs-cTnT assay and Abbott hs-cTnI assay, novel very high-sensitivity cTnI assays, such as the Nanosphere VeriSens and Quanterix SiMoA cTnI assays, have been developed. Additionally, a *Single Molecule Counting* (SMC) hs-cTnI assay developed by Singulex was briefly available for clinical use, and is the hs-cTnI assay utilized in the current study. The increased analytical sensitivity of these assays facilitates high precision cTn measurements which can be utilized for diagnostic purposes other than acute ischemia^11–13^.

With the advent of high-resolution computer tomography (CT) machines, Coronary Computed Tomography Angiography (CCTA) has become an attractive modality in the assessment of CAD. Population studies have demonstrated that an anatomical approach to evaluating chest pain is non-inferior to traditional cardiac stress testing and might enable targeted intervention and prevention of cardiovascular events^14,15^. In the evaluation of suspected chronic coronary syndromes (CCS), European guidelines (2019) emphasize the utility of CCTA^16^. Further, CCTA enables the quantification of coronary artery calcium (CAC) score, a robust CVD risk marker^17^. Additionally, international guidelines for prevention of CVD recommend both systematic and opportunistic application of multifactorial risk estimation scores to assess patient’s CVD risk prior to intervention^18,19^.

In this study we aimed to evaluate the diagnostic properties of hs-cTnI and hs-cTnT assays for obstructive CAD in patients with suspected CCS evaluated with CCTA. We hypothesized that (1) the utilization of a single hs-cTn measurement would enable rule-out of obstructive CAD, (2) the higher analytical sensitivity of the hs-cTnI assay would provide superior rule-out abilities to the hs-cTnT assay, and (3) the addition of hs-cTn to an established cardiovascular risk stratification model would enable more accurate identification of individuals at risk of obstructive CAD. Lastly (4), we aimed to assess the comparative association between hs-cTn and CAC scores in the study population.

## Material and methods

### Study design

This study utilizes data from a prospective cohort study from the University Hospital of North Norway (UNN), a secondary cardiological referral center, investigating the comparative performance of CCTA and invasive coronary angiography (clinical trial identifier: NCT01476579). A total of 1511 patients referred for invasive evaluation of CAD at the clinical discretion of designated cardiologists, were eligible for inclusion. Referral for coronary evaluation was independent of the current study and included classical sign and symptoms of CCS, such as exertional chest pain and dyspnea, evaluation of new onset heart failure without acute coronary syndrome, evaluation of primary arrythmias and evaluation of CAD prior to valve replacement surgery. Of the patients eligible for inclusion, 805 had missing data, declined participation or were otherwise unable to be included in the study or final analyses, or were excluded by predefined criteria. (Supplemental Fig. 1). The study was approved by the Norwegian Regional Committees for Medical and Health Research, Division North, and conducted in accordance with the Helsinki Declaration. Written informed consent was obtained from all participants.

### Cardiac computed tomography angiography

All CCTA were performed on a 128 × 2-slice dual source CT machine (Somatom Definition Flash, Siemens Medical Solutions, Erlangen, Germany) with the test protocol chosen based on patient heart rate characteristics.

All angiograms were analyzed at UNN by either of two senior thorax radiology consultants with > 5 years of experience and a Level 2 or equivalent expertise per the standards of the Society of Cardiovascular Computed Tomography^20^. Angiograms were described on a segmental basis per the American Heart Association classification^21^. CAC score was calculated using the Agatston method^22^.

The primary endpoint in this study was obstructive CAD, which was defined as the presence of any epicardial coronary luminal diameter reduction of 50% or more (CAD_50_) assessed by CCTA. Accordingly, patients with coronary stenosis ranging from 50% obstruction to complete occlusion were classified as CAD_50_. The presence of coronary plaques with < 50% stenoses was defined as ‘non-obstructive CAD’, and coronary arteries without plaques was defined as ‘no CAD’.

### Blood sampling and biochemical assays

Venous blood samples were obtained prior to the same-day CCTA examination. The samples were centrifuged, and the serum was frozen and stored at -80 °C at UNN. Analyses of cTnT was performed at the central clinical laboratory at UNN utilizing the Roche hs-cTnT assay (Elecsys STAT cardiac troponin T) on a Cobas 8000/e602 platform with a limit of detection (LoD) at 3 ng/L, as per manufacturer documentation, and a sex-neutral 99th percentile upper reference limit (URL) of 14 ng/L. The coefficient of variation (CV) was 2.8% at a sample concentration of 28 ng/L. cTnI was analyzed at Akershus University Hospital with the Singulex Clarity SMC ultra-sensitivity cTnI assay with a LoD at 0.08 ng/L and a sex-neutral 99th percentile URL of 6.74 ng/L, as reported by the manufacturer. In our laboratory the CV was 14.5% at a low concentration sample (0–2.0 ng/L, n = 60) and 6.8% at a high concentration sample (100.0 ng/L, n = 59).

To allow for an assumed hs-cTn distribution below the LoD, concentrations < 3 ng/L for hs-cTnT and < 0.08 ng/L for hs-cTnI are imputed as half the LoD in all analyses. To fully utilize the analytical precision of the hs-cTnI assay, concentrations are reported to one decimal place. hs-cTnT concentrations are reported as whole numbers, as recommended by the International Federation of Clinical Chemistry and Laboratory Medicine (IFCC).

### Cardiovascular risk stratification

European guidelines for CVD risk assessment in primary prevention currently utilize the Systematic Coronary Risk Estimation (SCORE) model, while the Norwegian national guidelines utilize the NORRISK2 model^18,23^. The NORRISK2 is a SCORE-like model calibrated to the demographic and morbidity characteristics of the Norwegian population. The NORRISK2 model calculates the age specific 10-year risk of fatal and non-fatal CVD events and yields both a graded and categorical evaluation of a subject’s risk profile. In the context of this article the NORRISK2 score is not used to provide pre-test probability scores for obstructive CAD or CVD prognostication, but rather as a graded marker of CVD risk without incorporating the associated age-dependent clinical decision limits^18,19,23,24^. Patient’s anginal burden is assessed by the Seattle Angina Questionnaire (SAQ)^25^.

### Statistical methods

All categorical variables are reported as absolute numbers with percentages and continuous variables as medians with quartiles 1 and 3. Baseline variables were analyzed with non-parametric tests; comparison of categorical variables was done using the Pearson’s chi-square test and continuous variables with the Mann–Whitney-*U* test. Spearman's rank correlation and linear regression analyses were utilized to assess predictors of hs-cTn. Logistic and linear regression models were used to analyze associations between hs-cTn and the presence of CAD and CAC scores, respectively. The following covariates were a priori selected and included in the regression models as adjustment for known confounders: Age, sex, current smoking, a history of CAD, diabetes or heart failure (HF), body mass index (BMI), systolic blood pressure (SBP), low density lipoprotein cholesterol (LDL-C) and estimated glomerular filtration rate (eGFR). All covariates were first assessed for univariable associations, followed by multivariable modelling of significant predictors utilizing a stepwise backward elimination approach. Due to right-skewed distributions, we use base 2 log-transformed values of hs-cTn and LDL-C in all regression models. CAC scores of zero was imputed as 0.1 and log_2_-transformed in all models. The continuous association between CAC scores and hs-cTn was assessed by flexible cubic spline models tested for best fit (2 to 7 knots) based on the lowest Akaike Information Criterion. Patients were stratified by tertiles of the NORRISK2-score as either low, intermediate or high-risk. The performance of hs-cTn in diagnosing CAD_50_ was examined by receiver operating characteristics curves (ROC) with corresponding c-statistics, continuous Net Reclassification Improvement (cNRI) and Integrated Discrimination Improvement (IDI).

Sensitivity, specificity and predictive values were calculated for the hs-cTn concentration thresholds at the assay specific LoD, total population median and the 99th percentile URLs. To facilitate comparison between the hs-cTnI and hs-cTnT assays in individuals with very low hs-cTn concentrations, the group-specific hs-cTnI 25th, 50th and 75th concentration percentiles were used as diagnostic thresholds in the subgroup of patients with hs-cTnT concentrations below the LoD.

All statistical analyses were performed with STATA 15 (StataCorp. 2017. *Stata Statistical Software: Release 15*.*1.* College Station, TX: StataCorp LLC). Two-sided *p*-values with a significance level of 5% or confidence intervals (CI) with a confidence level of 95% are used to indicate assumed statistical significance.

## Results

### Baseline characteristics

In total, 706 patients referred for angiographic evaluation of CAD were included in the study analyses. Where available, the primary reason for referral was suspected CCS in 645 (91%) patients, evaluation of CAD prior to valve replacement surgery in 31 (4%) patients and new onset or worsened HF in 14 (2%) patients. Median age was 65 (range 28–87) years and 245 (35%) were women. Established CAD and HF were present in 280 (40%) and 41 (6%) patients. An overview of patient characteristics by primary reason for referral is available as Supplemental Table 6.

CAD_50_ was present in 397 (56%) patients, 233 (33%) had non-obstructive CAD and 76 (11%) had no CAD. The prevalence of CAD_50_ in patients referred for evaluation of new onset or worsened HF or evaluation of CAD prior to valve replacement surgery was 36% and 58%, respectively (Supplemental Table 1). Patients with CAD_50_ were older, more often male, had higher BMI, higher systolic blood pressure, lower eGFR, lower LDL-C, had a higher comorbidity burden and used more preventive medication. CAC scores were higher in patients *with* versus without CAD_50_: median 501 (Q1-3 145–1427) and 23 (0–201), respectively. The median (Q1-3) NORRISK2 score was 13 (8–18) in patients with CAD_50_ and 9 (5–13) in patients without CAD_50_ (*p* < 0.001). (Table 1).

**Table 1:** Baseline characteristics of patients referred for coronary computed tomography angiography stratified by the presence of ≥ 50% luminal stenosis (CAD_50_) in any coronary segment.

	CAD_50_ negative (n = 309)	CAD_50_ positive (n = 397)	*p* value
Age, years	62 (55, 69)	66 (59, 73)	< 0.001
Female sex (%)	137 (44%)	108 (27%)	< 0.001
Family history of ischemic heart disease (%)	205 (66%)	261 (66%)	0.87
Current smoker (%)	62 (20%)	65 (16%)	0.21
Body Mass Index (kg/m^2^)	27 (25, 30)	28 (26, 31)	0.012
**History of**			
Hypertension (%)	152 (49%)	259 (66%)	< 0.001
Diabetes (%)	46 (15%)	100 (25%)	< 0.001
Coronary heart disease (%)	64 (20%)	226 (55%)	< 0.001
Heart failure (%)	17 (6%)	24 (6%)	0.75
**Medication**			
Acetylsalisylic acid (%)	202 (66%)	334 (84%)	< 0.001
Statins (%)	185 (60%)	317 (80%)	< 0.001
Beta blocker (%)	161 (52%)	271 (68%)	< 0.001
Calcium channel blocker (%)	48 (16%)	99 (25%)	0.002
ACE inhibitor (%)	42 (14%)	83 (21%)	0.012
Angiotensin II receptor blocker (%)	70 (23%)	120 (30%)	0.025
Diuretic (%)	60 (20%)	120 (30%)	0.002
Warfarin (%)	28 (9%)	39 (10%)	0.73
Insulin (%)	14 (5%)	24 (6%)	0.38
Other anti-diabetics (%)	31 (10%)	60 (15%)	0.046
Systolic blood pressure, mmHg	140 (128, 155)	146 (134, 161)	0.002
eGFR ckd-epi, ml/min/1.73m^2^	89 (80, 98)	85 (74, 94)	< 0.001
Coronary artery calcium score	23 (0, 201)	501 (145, 1427)	< 0.001
NORRISK2 score	9 (5, 13)	13 (8, 18)	< 0.001
Low density lipoprotein cholesterol, mmol/L	3.0 (2.3, 3.7)	2.7 (2.2, 3.3)	0.003

Presented as absolute numbers or medians with percentages or quartiles 1 and 3 (Q1-3). *p* values are for between-group differences. CAD_50 _- ≥ 50% luminal stenosis in any coronary segment, eGFR ckd-epi – estimated glomerular filtrations rate using the Chronic Kidney Disease Epidemiology Collaboration equation, NORRISK2 score – Norwegian calibrated cardiovascular risk estimate.

### Predictors of high sensitivity cardiac troponin I and T

All patients had hs-cTnI concentrations above the LoD (median 1.9 [Q1-3 1.2–3.6] ng/L), whereas 511 patients (72%) had hs-cTnT above the LoD (median 5 [2–11] ng/L). The correlation between hs-cTnI and hs-cTnT was 0.64, *p* < 0.001.

The median (Q1-3) hs-cTnI concentrations in women and men were 1.4 (0.8–2.2) ng/L and 2.3 (1.4–4.8) ng/L (*p* < 0.001). The respective median hs-cTnT concentrations were 4 (2–8) ng/L and 8 (4–13) ng/L (*p* < 0.001).

In adjusted linear regression modelling of the total population, older age, male sex, history of diabetes, history of HF and higher BMI significantly predicted both higher hs-cTnI and hs-cTnT concentrations. Lower eGFR was significantly associated with higher hs-cTnT but not hs-cTnI, whereas higher SBP was significantly associated with higher hs-cTnI, but not hs-cTnT. These variables accounted for 33% and 29% of the variance in hs-cTnI and hs-cTnT in the regression models (adjusted *R*^2^ = 0.33 and 0.29). (Supplemental Table 2).

### Association between cardiac troponins and obstructive CAD

The median (Q1-3) hs-cTnI concentration was 2.3 (1.4–4.7) ng/L in patients with CAD_50_, 1.5 (1.0–2.6) ng/L in patients with non-obstructive CAD, and 1.3 (0.8–2.4) ng/L in patients without CAD, *p* < 0.001 for both comparisons. The corresponding values for hs-cTnT were 8 (3–12) ng/L, 6 (2–9) ng/L and 5 (2–8) ng/L, *p* < 0.001 for both comparisons.

Men with CAD_50_ had higher hs-cTn concentrations than women with CAD_50_ (hs-cTnI: 2.7 [1.6–5.2] ng/L vs. 1.6 [1.0–2.4] ng/L, *p* < 0.001, and hs-cTnT: 8 [4–14] ng/L vs. 5 [2–9] ng/L, *p* < 0.001).

Higher concentrations of hs-cTnI and hs-cTnT were associated with CAD_50_ in unadjusted analyses (hs-cTnI: Odds Ratio (OR) 1.45, 95% CI [1.28–1.64], *p* < 0.001, hs-cTnT: OR 1.27 [1.13–1.41], *p* < 0.001). However, after adjusting for age, sex, smoking, history of CAD, diabetes and HF, BMI, SBP, LDL-C and eGFR, only hs-cTnI remained significantly associated with CAD_50_ (OR 1.20 [1.05–1.38], *p* = 0.009). (Supplemental Table 3).

The area under the ROC curve (ROC-AUC) for hs-cTnI and hs-cTnT in predicting CAD_50_ was 0.65 (0.61–0.69) and 0.60 (0.65–0.64), respectively, *p* = 0.01 for difference. There were no significant sex-dependent differences between the AUCs of hs-cTnI and hs-cTnT in diagnosing CAD_50_ (hs-cTnI: Women: 0.60 [0.53–0.67], Men: 0.63 [0.58–0.68], *p* = 0.53; hs-cTnT: Women: 0.55 [0.48–0.62], Men: 0.60 [0.54–0.65], *p* = 0.32).

### Diagnostic properties of cardiac troponins stratified by risk categories

**Figure 1:**
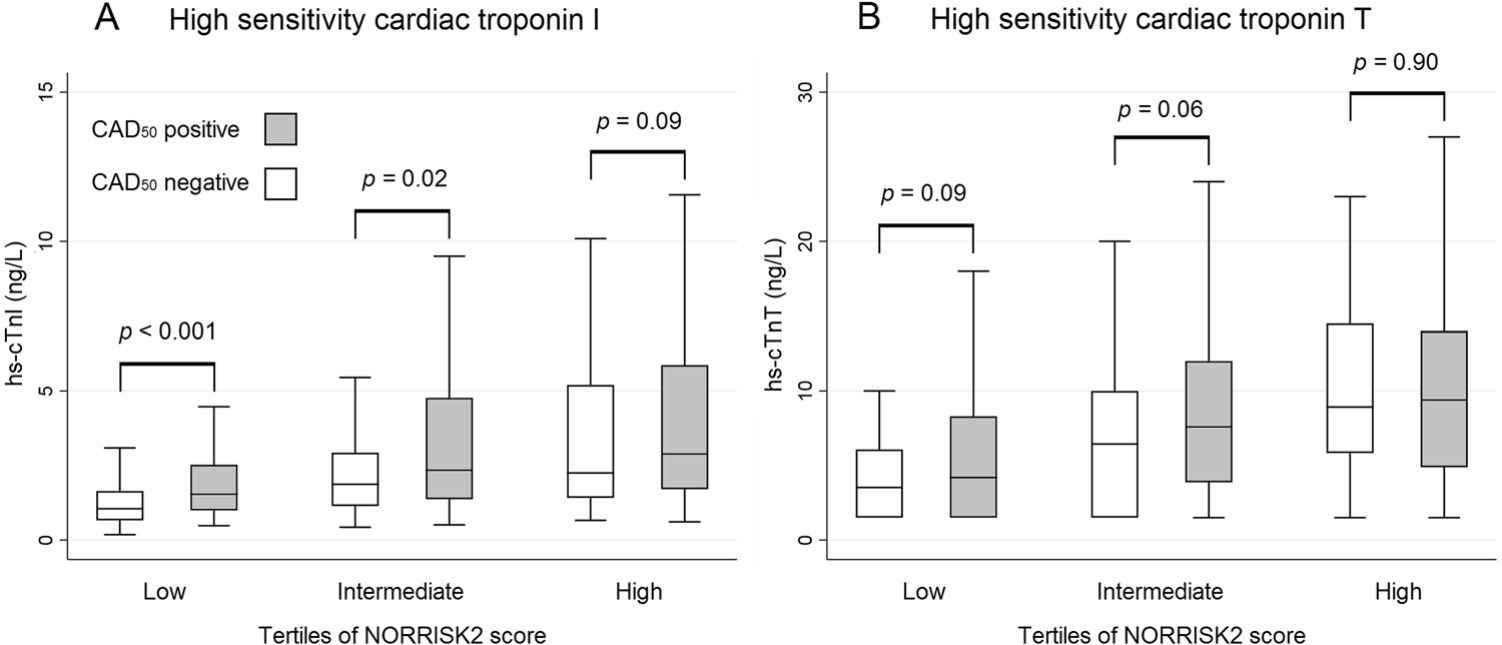
Boxplot with whiskers showing concentrations of cardiac troponin I (Panel **A**) and cardiac troponin T (Panel **B**) in patients with and without obstructive coronary artery disease, stratified by tertiles of NORRISK2 score. CAD_50_ – obstructive coronary artery disease, hs-cTnI – high-sensitivity cardiac troponin I, hs-cTnT – high-sensitivity cardiac troponin T.

The NORRISK2-score was computable in 696 of the 706 patients included in the main analyses. CAD_50_ was present in 96 (41%), 127 (55%) and 168 (72%) in the low, intermediate and high NORRISK2 tertiles. The median (Q1-3) hs-cTnI concentrations were 1.2 (0.8–1.9) ng/L, 2.2 (1.3–3.8) ng/L and 2.7 (1.6–5.6) ng/L, respectively. The corresponding values for hs-cTnT were 4 (2–7) ng/L, 7 (3–11) ng/L and 9 (5–14) ng/L. (Fig. 1). The median (Q1-3) summary SAQ-scores in the same groups were 72 (56–89), 69 (53–83) and 65 (50–86), respectively. SAQ-scores were not significantly associated with CAD_50_ in any of the tertiles in univariable logistic regression analyses.

In adjusted logistic regression modelling, hs-cTnI was associated with CAD_50_ in the lowest risk tertile (OR 1.52, 95% CI [1.19–1.93], *p* = 0.001). hs-cTnT concentrations were not significantly associated with CAD_50_ in the univariable analysis (OR 1.16 [0.95–1.43], *p* = 0.15). In the intermediate risk tertile, hs-cTnI was associated with CAD_50_ in univariable analyses (OR 1.23, 95%CI [1.01–1.48], *p* = 0.04). This association was attenuated and statistically not significant in adjusted analyses. hs-cTnT was not significantly associated with CAD_50_ in univariable analyses (OR 1.21 [0.99–1.48], *p* = 0.06). In the highest risk tertile, neither hs-cTnI nor hs-cTnT were associated with CAD_50_ in univariable analyses (hs-cTnI: OR 1.25, 95%CI [0.99–1.58], *p* = 0.06; hs-cTnT: OR 1.00 [0.80–1.26], *p* = 0.98). (Supplemental Table 4).

Sensitivity, specificity, positive predictive values (PPV) and negative predictive values (NPV) for the LoD, total population cTn medians and the assay specific 99th percentile URL stratified by risk tertiles are presented in Table 2. For both hs-cTnI and hs-cTnT, the highest specificity and NPV, and lowest sensitivity and PPV were observed in the lowest risk tertile. The diagnostic performance of hs-cTnI concentrations in the subgroup of patients with hs-cTnT concentrations below the LoD are presented in Supplemental Table 5.

**Table 2:** Sensitivity, specificity, positive and negative predictive values for the high-sensitivity cardiac troponin cut-offs by risk strata.

cTn cut-off			Total population (n = 696)		Low risk (n = 232)		Intermediate risk (n = 232)		High risk (n = 232)	
			% (95% CI)	Patients with cTn > cut-off (% of group)	% (95% CI)	Patients with cTn > cut-off (% of group)	% (95% CI)	Patients with cTn > cut-off (% of group)	% (95% CI)	Patients with cTn > cut-off (% of group)
LoD	hs-cTnI	*Sensitivity*	NA	696 (100)	NA	232 (100)	NA	232 (100)	NA	232 (100)
		*Specificity*	NA		NA		NA		NA	
		*PPV*	NA		NA		NA		NA	
		*NPV*	NA		NA		NA		NA	
	hs-cTnT	*Sensitivity*	76 (72–80)	504 (72)	56 (46–66)	127 (55)	80 (72–87)	178 (77)	85 (78–90)	200 (86)
		*Specificity*	32 (27–38)		46 (38–55)		28 (19–37)		9 (4–19)	
		*PPV*	59 (55–63		43 (34–52)		57 (50–65)		71 (64–77)	
		*NPV*	51 (44–59)		60 (50–69)		54 (40–67)		19 (7–36)	
Total population median	hs-cTnI	*Sensitivity*	59 (54–64)	348 (50)	34 (25–45)	60 (26)	64 (55–72)	134 (58)	70 (63–77)	154 (66)
		*Specificity*	62 (56–67)		79 (72–86)		50 (40–60)		44 (31–57)	
		*PPV*	66 (61–71)		53 (40–66)		60 (52–69)		77 (69–83)	
		*NPV*	54 (49–59)		63 (55–70)		53 (43–63)		36 (25–48)	
	hs-cTnT	*Sensitivity*	58 (53–63)	350 (50)	34 (25–45)	62 (27)	61 (52–70)	130 (56)	68 (60–75)	158 (68)
		*Specificity*	59 (53–65)		79 (71–85)		51 (41–60)		31 (20–44)	
		*PPV*	64 (59–69)		53 (40–66)		60 (51–69)		72 (65–79)	
		*NPV*	52 (47–57)		63 (55–70)		52 (42–62)		27 (17–39)	
URL	hs-cTnI	*Sensitivity*	17 (13–21)	91 (13)	8 (4–16)	12 (5)	17 (11–24)	32 (14)	23 (17–30)	47 (20)
		*Specificity*	92 (88–95)		96 (92–99)		90 (82–95)		86 (75–93)	
		*PPV*	73 (42–50)		62 (32–86)		66 (47–81)		81 (67–91)	
		*NPV*	46 (42–50)		60 (53–66)		47 (40–54)		30 (23–37)	
	hs-cTnT	*Sensitivity*	20 (16–25)	112 (16)	8 (4–16)	13 (6)	19 (13–27)	34 (15)	28 (21–35)	65 (28)
		*Specificity*	89 (85–92)		96 (92–99)		91 (83–95)		72 (59–82)	
		*PPV*	71 (61–79)		62 (32–86)		71 (53–85)		72 (60–83)	
		*NPV*	47 (43–51)		60 (53–66)		48 (41–55)		28 (23–36)	
		*ROC-AUC (hs-cTnI)*	65 (60–69)		64 (57–71)		59 (52–66)		57 (49–66)	
		*ROC-AUC (hs-cTnT)*	60 (56–64)		56 (49–64)		57 (50–64)		51 (43–59)	

hs-cTnI – high-sensitivity cardiac troponin I, hs-cTnT – high-sensitivity cardiac troponin T, PPV – positive predictive value, NPV – negative predictive value, ROC-AUC – area under the receiver operating characteristics curve.

The ROC-AUC for the NORRISK2-score in predicting CAD_50_ in the total population was 0.65 (0.61–0.69). Neither hs-cTnI, nor hs-cTnT significantly improved the AUC for predicting CAD_50_. The addition of hs-cTnI to the NORRISK2-score significantly reclassified patients to a more correct risk stratum, mainly by downgrading the risk attributed to patients without CAD_50_ (cNRI 0.28 95% CI [0.11–0.42], IDI 0.02 [0.005–0.05]). The addition of hs-cTnT to the NORRISK2-score did not yield significant reclassification of risk (cNRI 0.12 [-0.13–0.25]), IDI 0. 004 [-0.002–0.02]). (Supplemental Table 6).

### Association between hs-cTn and coronary artery calcium

**Figure 2:**
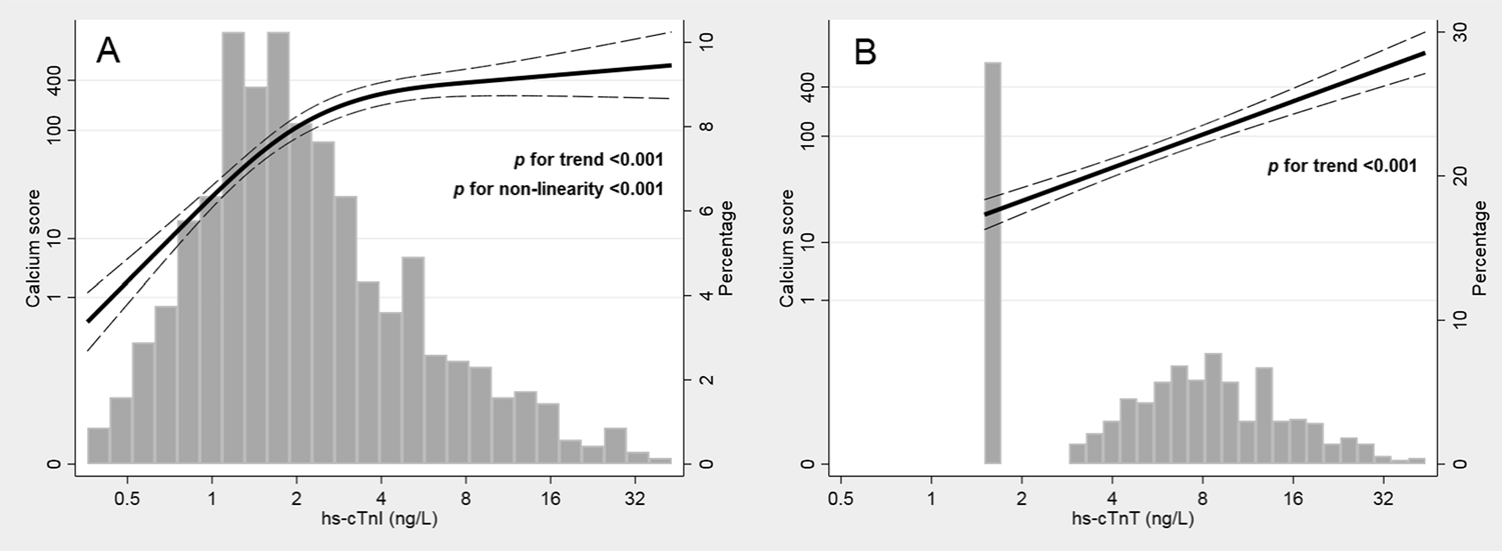
Restricted cubic splines models showing the association between log_2_-transformed calcium score and log_2_-transformed cardiac troponin I (Panel **A**) and T (Panel **B**) concentrations in the total population with available calcium score (n = 646). The splines are superimposed on histograms of cardiac troponin I and T distributions. The logarithmic x and y-axis have been exponentiated. The hs-cTnI-model is fitted to three knots and the hs-cTnT-model to two knots, based on the best fit according to the lowest Akaike Information Criterion. The dotted lines reflect the 95% confidence interval. hs-cTnI – high-sensitivity cardiac troponin I, hs-cTnT – high-sensitivity cardiac troponin T.

CAC scores were available in 646 patients (median 209 [Q1-3 15–769]). In patients referred for evaluation of new onset or worsened HF or evaluation of CAD prior to valve replacement surgery the CAC scores were median 339 (Q1-3 59–842) and 530 (104–1534), respectively. There was a graded association between higher concentrations of hs-cTnI and hs-cTnT and higher CAC-score in the total population (*p* < 0.001 for overall trend for both; Fig. 2). This association was linear for hs-cTnT (Panel **B**) and non-linear for hs-cTnI (Panel **A**) with a stronger association below 3 ng/L. Log_2_-transformed hs-cTn significantly predicted log_2_-transformed CAC scores in adjusted linear regression analyses (hs-cTnI: *B* 0.68, 95% CI [0.43–0.93], *p* < 0.001; hs-cTnT: *B* 0.52, 95% CI [0.25–0.79], *p* < 0.001).

## Discussion

Our study of hs-cTnI and hs-cTnT in diagnosing obstructive CAD in patients with suspected CCS has three main findings. First, there was a graded association between higher concentrations of circulating hs-cTn and the severity of CAD in the total population, both assessed by CAC and the presence of stenotic coronary lesions. The relationship between hs-cTnI, but not hs-cTnT, and obstructive CAD remained significant after adjusting for confounders. Second, higher hs-cTn concentrations and higher NORRISK2-scores were both associated with obstructive CAD and with comparable discriminatory performance. The diagnostic properties of hs-cTn were dependent on the patients’ background CVD risk as assessed by the NORRISK2-score, and hs-cTnI provided some reclassification abilities, mainly by downgrading the risk attributed to patients without obstructive CAD. Third, low concentrations of both hs-cTnI and hs-cTnT provide some rule-out ability of obstructive CAD in patients with low background CVD risk, albeit with modest specificity and NPV.

### Cardiac troponin and the presence of coronary stenosis in chronic coronary syndrome

The association between cTn and CAD in CCS has previously been demonstrated. In the Prospective Multicenter Imaging Study for Evaluation of Chest Pain (PROMISE) trial, investigators observed a graded relationship between higher concentrations of hs-cTnI, measured with a SMC hs-cTnI assay, and the severity and extent of coronary lesions assessed with CCTA^26^. In the Scottish Computed Tomography of the Heart (SCOT-HEART) trial, investigators found that atherosclerotic burden, as well as left ventricular mass, were independent predictors of increased hs-cTnI concentrations measured with a SMC assay^27^. Similar association between anatomical severity of CAD and concentrations of circulating cTn has been confirmed in other smaller studies with coronary imaging^28,29^.

When assessing functional outcomes of CAD, higher concentrations of hs-cTnT and hs-cTnI have been shown to be associated with reversible myocardial ischemia^30^, even after adjusting for cardiac structure and function^31^. In the Evaluation of Integrated Cardiac Imaging (EVINCI) trial, patients with suspected CCS underwent both an anatomical assessment of CAD *and* a functional imaging test, and abnormalities in either test were independently associated with higher hs-cTnT concentrations^32^. In the setting of CCS, the observed concentrations of cTn are often minute compared to those observed in AMI, and often below the LoD of the contemporary cTn assays employed. This clearly limits the diagnostic efficacy of contemporary cTn assays, as the analytical noise caused by high CV at these concentrations limits our ability to correctly discriminate patients. Whether higher sensitivity assays with increased analytical precision at low concentrations might increase the diagnostic yield in patients with the lowest cTn concentrations has not been thoroughly examined. In the PROMISE trial, the investigators were able to measure hs-cTnI concentrations with high precision in 98.5% of the study population^26^. This, however, did not translate into a clinically viable rule-out model for CAD. As diagnostic performance is both test *and* population dependent, we extend these results to a higher risk population referred for invasive coronary angiography (CAD_50_ present in 56% compared to 24% in PROMISE), as well as compare the performance of high-sensitivity assays for both the I and T isotype. We further stratify patient by CVD risk with the SCORE-like NORRISK2 model, calibrated to account for national demographic and comorbidity idiosyncrasies. In the majority of the population, hs-cTnI and hs-cTnT had comparable diagnostic performance. As expected, the NPV of cTn for ruling out obstructive CAD was greatest in patients with the lowest CVD risk. In contrast, cTn did not sufficiently discriminate between those with and without obstructive CAD in higher risk patients. In the subgroup of patients with hs-cTnT concentrations below the LoD, the superior analytical sensitivity of hs-cTnI extended the diagnostic abilities of cTn and enabled further discrimination of patients, albeit with high statistical uncertainty. However, even with the superior analytical sensitivity, hs-cTnI did not provide clinically satisfactory predictive values and the diagnostic yield in this group was low.

### Mechanisms of elevated cardiac troponin in coronary artery disease

cTn can be chronically elevated in conditions with ongoing myocardial injury such as CCS and HF and chronic kidney disease. Several mechanisms have been proposed, both ischemic and non-ischemic, but the exact pathophysiological processes underlying these phenomena are not fully understood^33^. Indeed, most risk factors for CVD are associated with increased cTn concentrations^2^. As such, patients with a higher baseline CVD risk, defined by comorbidity burden, are more likely to have CAD-independent chronically elevated cTn concentrations. Thus, the contribution of chronic ischemia to the circulating cTn concentration is less important, and this may explain the limited diagnostic performance of hs-cTn in patients with higher CVD risk, observed in our study. Additionally, only hs-cTnI was associated with obstructive CAD in models adjusted for demographics and comorbidities and might indicate differential confounding by these factors. Indeed, in a study by Welsh et al. both hs-cTnI and hs-cTnT were associated with CVD risk in the general population^34^. In this study, only hs-cTnI was associated with CAD, while only hs-cTnT was associated with risk of non-CVD death. In another study, by Omland et al., hs-cTnI was associated with incidence of myocardial infarction, whereas hs-cTnT was not^6^. As in our data, the differential associations are subtle, and further research is needed, but they nonetheless strengthens the assumption that the pathophysiological determinants of increased concentrations of hs-cTn are isotype-dependent, and that hs-cTnI might be superior to hs-cTnT as a marker of CAD.

### Clinical applicability of high sensitivity cardiac troponin in risk assessment

In the total study population, both hs-cTnI and hs-cTnT displayed diagnostic performance comparable to the NORRISK2 score. Although there was no significant change in the ROC-AUC, adding hs-cTnI to the NORRISK2 score reclassified patients to a more correct risk stratum, mainly by downgrading the risk attributed to patients *without* obstructive CAD. hs-cTnT, however, did not display any significant reclassification ability. This is possibly explained by the higher analytical sensitivity of the hs-cTnI assay, enabling discrimination of patients with very low cTn concentrations. In the comparable SCOT-HEART trial, hs-cTnI measured with the same SMC assay displayed similar reclassification abilities when added to the CAD Consortium score for pre-test probability of obstructive CAD, mainly by downgrading the risk attributed to intermediate and high-risk patients^35^. Thus, a single measurement of hs-cTn may provide clinically important information to the treating physician, not only with respect to prognosis^36^, but also diagnosis in CCS. Whether hs-cTnI is superior to hs-cTnT in this regard remains to be conclusively determined.

As cTn concentrations are heavily dependent on comorbidities and disease characteristics, the diagnostic and prognostic efficacy of cTn exists on a continuum and is less robustly assessed by an arbitrary cut-off. Whether a singular diagnostic threshold or a weighted continuous approach is utilized, care should be taken to integrate confounding information when developing clinical decision tools. With increasing assay sensitivity and the application of cTn in conditions other than ACS, the limited disease specificity and confounded nature of cTn will become more evident and educational efforts should be made to train physicians to correctly interpret cTn concentrations in any given clinical context.

## Limitations

As all patients eligible for inclusion were initially referred for invasive evaluation of CAD and symptoms suggestive of CCS, the study population had enriched risk of CAD, and indeed the prevalence of obstructive CAD in this study was high. There was a substantial loss of patients eligible for inclusion in the study, and as such the introduction of a selection bias cannot be discounted or controlled for. It is, however, reasonable to assume that the prevalence of CAD in an unselected population would be significantly lower. Consequently, our results might be skewed, and the real-world efficacy of hs-cTn at the various cut-offs might differ from the results of this study. We attempt to control for this population dependence by adjusting for known confounders and stratifying patients based on the multi-factorial NORRISK2 risk score. Current guidelines recommend the application of pre-test probability estimation in the diagnosis of CCS. Our utilization of the NORRISK2-score does not formally provide this information, as its intended utilization is as a prognostic tool in a primary prevention setting. However, traditional CVD risk factors have demonstrated independent discriminatory abilities for obstructive CAD in the setting of CCS^37^. Our intended use of the NORRISK2-score is to give a graded estimate of patient’s CVD risk based on its incorporation of these traditional risk factors. Further, we do not have access to any longitudinal outcome data for the patients in the study. This limits our ability to assess any clinical significance resulting from using hs-cTn as a decision tool in the context of CCS. Finally, we do not have data on cardiac structure and function and therefore cannot accurately estimate the impact of myocardial remodeling on circulating hs-cTn.

## Conclusion

Elevated concentrations of hs-cTnI and hs-cTnT are associated with obstructive CAD and higher CAC burden. Our results suggest that hs-cTn have diagnostic value in patients with low baseline CVD risk, while the diagnostic value of hs-cTn measurements in patients with higher risk seems limited. hs-cTnI appears to have superior diagnostic properties to hs-cTnT with regard to CAD, and the added analytical sensitivity of the hs-cTnI assay add discriminatory power in patients with very low hs-cTn concentrations. Future studies randomizing CCS patients to troponin-guided decision-making or standard care, with long term follow-up, is needed.

## Supplementary Information

Supplementary Information.

## References

[1] Garg P (2017). Cardiac biomarkers of acute coronary syndrome: from history to high-sensitivity cardiac troponin. Int. Emerg. Med..

[2] Kelley WE, Januzzi JL, Christenson RH (2009). Increases of cardiac troponin in conditions other than acute coronary syndrome and heart failure. Clin. Chem..

[3] Saunders JT (2011). Cardiac troponin T measured by a highly sensitive assay predicts coronary heart disease, heart failure, and mortality in the atherosclerosis risk in communities study. Circulation.

[4] deFilippi, C. R. *et al.* Association of serial measures of cardiac troponin T using a sensitive assay with incident heart failure and cardiovascular mortality in older adults. *JAMA* **304**, 2494-2502, doi:10.1001/jama.2010.1708 (2010) 10.1001/jama.2010.1708 PMC3559101 21078811

[5] de Lemos JA (2010). Association of troponin T detected with a highly sensitive assay and cardiac structure and mortality risk in the general population. JAMA.

[6] Omland T (2013). Prognostic value of cardiac troponin I measured with a highly sensitive assay in patients with stable coronary artery disease. J. Am. Coll. Cardiol..

[7] Than MP (2018). Detectable high-sensitivity cardiac troponin within the population reference interval conveys high 5-year cardiovascular risk: an observational study. Clin. Chem..

[8] Ndrepepa G (2011). Comparison of prognostic value of high-sensitivity and conventional troponin T in patients with non-ST-segment elevation acute coronary syndromes. Clin. Chim. Acta.

[9] Sandoval Y (2019). Clinical features and outcomes of emergency department patients with high-sensitivity cardiac troponin i concentrations within sex-specific reference intervals. Circulation.

[10] Apple FS (2015). IFCC educational materials on selected analytical and clinical applications of high sensitivity cardiac troponin assays. Clin. Biochem..

[11] Rissin DM (2010). Single-molecule enzyme-linked immunosorbent assay detects serum proteins at subfemtomolar concentrations. Nat. Biotechnol..

[12] Wilson SR (2009). Detection of myocardial injury in patients with unstable angina using a novel nanoparticle cardiac troponin I assay: observations from the PROTECT-TIMI 30 Trial. Am. Heart J..

[13] Todd J (2007). Ultrasensitive flow-based immunoassays using single-molecule counting. Clin. Chem..

[14] Investigators S-H (2018). Coronary CT Angiography and 5-Year Risk of Myocardial Infarction. N. Engl. J. Med..

[15] Douglas PS (2015). Outcomes of anatomical versus functional testing for coronary artery disease. N. Engl. J. Med..

[16] Knuuti J (2019). ESC Guidelines for the diagnosis and management of chronic coronary syndromes. Eur. Heart J..

[17] Greenland P, Blaha MJ, Budoff MJ, Erbel R, Watson KE (2018). Coronary calcium score and cardiovascular risk. J. Am. Coll. Cardiol..

[18] Piepoli MF (2016). 2016 European Guidelines on cardiovascular disease prevention in clinical practice: The Sixth Joint Task Force of the European Society of Cardiology and Other Societies on Cardiovascular Disease Prevention in Clinical Practice (constituted by representatives of 10 societies and by invited experts)Developed with the special contribution of the European Association for Cardiovascular Prevention & Rehabilitation (EACPR). Eur. Heart J..

[19] Goff DC (2014). 2013 ACC/AHA guideline on the assessment of cardiovascular risk: a report of the American College of Cardiology/American Heart Association Task Force on Practice Guidelines. Circulation.

[20] Budoff MJ (2005). ACCF/AHA clinical competence statement on cardiac imaging with computed tomography and magnetic resonance: a report of the American College of Cardiology Foundation/American Heart Association/American College of Physicians Task Force on Clinical Competence and Training. J. Am. Coll. Cardiol..

[21] Austen, W. G. *et al.* A reporting system on patients evaluated for coronary artery disease. Report of the Ad Hoc Committee for Grading of Coronary Artery Disease, Council on Cardiovascular Surgery, American Heart Association. *Circulation* **51**, 5–40, doi:10.1161/01.cir.51.4.5 (1975). 10.1161/01.cir.51.4.5 1116248

[22] Agatston AS (1990). Quantification of coronary artery calcium using ultrafast computed tomography. J. Am. Coll. Cardiol..

[23] Helsedirektoratet. *Forebygging av hjerte- og karsykdom. Nasjonal faglig retningslinje for forebygging av hjerte- og karsykdom*, https://helsedirektoratet.no/retningslinjer/forebygging-av-hjerte-og-karsykdom (2017).

[24] Selmer R (2017). NORRISK 2: A Norwegian risk model for acute cerebral stroke and myocardial infarction. Eur. J. Prev. Cardiol..

[25] Spertus JA (1995). Development and evaluation of the Seattle Angina Questionnaire: a new functional status measure for coronary artery disease. J. Am. Coll. Cardiol..

[26] Januzzi JL (2019). High-sensitivity troponin i and coronary computed tomography in symptomatic outpatients with suspected CAD: insights from the PROMISE trial. JACC Cardiovasc. Imaging.

[27] Bing R (2019). Clinical determinants of plasma cardiac biomarkers in patients with stable chest pain. Heart (Br. Cardiac. Soc.).

[28] Laufer EM (2010). The extent of coronary atherosclerosis is associated with increasing circulating levels of high sensitive cardiac troponin T. Arterioscler. Thromb. Vasc. Biol..

[29] Reckord N (2017). High sensitivity troponin I and T reflect the presence of obstructive and multi-vessel coronary artery disease being assessed by coronary computed tomography angiography. Curr. Pharm. Biotechnol..

[30] Sabatine MS, Morrow DA, de Lemos JA, Jarolim P, Braunwald E (2009). Detection of acute changes in circulating troponin in the setting of transient stress test-induced myocardial ischaemia using an ultrasensitive assay: results from TIMI 35. Eur. Heart J..

[31] Myhre PL (2018). Cardiac troponin T concentrations, reversible myocardial ischemia, and indices of left ventricular remodeling in patients with suspected stable angina pectoris: a DOPPLER-CIP substudy. Clin. Chem..

[32] Caselli C (2016). Effect of coronary atherosclerosis and myocardial ischemia on plasma levels of high-sensitivity troponin T and NT-proBNP in patients with stable angina. Arterioscler. Thromb. Vasc. Biol..

[33] Mair J (2018). How is cardiac troponin released from injured myocardium?. Eur. Heart J. Acute Cardiovasc. Care.

[34] Welsh P (2019). Cardiac TROPONIN T and Troponin I in the general population. Circulation.

[35] Adamson PD (2018). High-sensitivity cardiac Troponin I and the diagnosis of coronary artery disease in patients with suspected angina pectoris. Circ. Cardiovasc. Qual. Outcomes.

[36] Omland T (2009). A sensitive cardiac troponin T assay in stable coronary artery disease. N. Engl. J. Med..

[37] Patel MR (2010). Low diagnostic yield of elective coronary angiography. N. Engl. J. Med..

