# Supplementary material for: Cardiac troponin I and T for ruling out coronary artery disease in suspected chronic coronary syndrome
Source: Sci Rep. 2022 Jan 18;12:945. doi: 10.1038/s41598-022-04850-7 (PMC8766564; doi:10.1038/s41598-022-04850-7)
Supplement: Supplementary file 1 — Supplementary Information. [file 41598_2022_4850_MOESM1_ESM.docx]

**Cardiac troponin I and T for ruling out coronary artery disease in suspected chronic coronary syndrome**

**Supplemental material**

**Supplemental Table 1**. Characteristics of patients referred for evaluation of coronary artery disease by referral indication

|  | **n (%)** | **Female sex n (%)** | **Age (median, Q1-Q3)** | **Dyspnoea prior to referral n (%)** | **CAC-score  (median, Q1-Q3)** | **CAD_50_  n (%)** |
| --- | --- | --- | --- | --- | --- | --- |
| Chronic coronary syndrome | 645 (91) | 229 (36) | 64 (56-71) | 395 (61) | 203 (14-759) | 367 (57) |
| New onset heart failure | 14 (2) | 5 (36) | 66 (58-69) | 12 (86) | 339 (59-842) | 5 (36) |
| Valvular disease | 31 (4) | 10 (32) | 72 (66-76) | 28 (90) | 530 (104-1534) | 18 (58) |
| Arrythmia | 9 (1) | 0 (0) | 64 (57-66) | 6 (67) | 93 (0-360) | 4 (44) |
| Other | 7 (1) | 1 (14) | 57 (53-65) | 2 (29) | 39 (0-1898) | 3 (43) |

*Abbreviations: CAC-score – coronary artery calcium score, CAD_50_ – obstructive coronary artery disease*

**Supplemental Table 2.** Predictors of high-sensitivity cardiac troponin I and T in unadjusted and adjusted linear regression models in the total population. Covariates with a p-value <0.05 for the association in the univariable model were included in the multivariable model.

|  | **Cardiac troponin I** | | | | | | **Cardiac troponin T** | | | | | |
| --- | --- | --- | --- | --- | --- | --- | --- | --- | --- | --- | --- | --- |
|  | **Univariable analyses** | | | **Multivariable analyses** | | | **Univariable analyses** | | | **Multivariable analyses** | | |
|  | **B** | **95% Confidence interval** | **p-value** | **B** | **95% Confidence interval** | **p-value** | **B** | **95% Confidence interval** | **p-value** | **B** | **95% Confidence interval** | **p-value** |
| Age (years) | 0.55 | 0.45 - 0.64 | <0.001 | 0.50 | 0.41 - 0.59 | <0.001 | 0.53 | 0.43 - 0.62 | <0.001 | 0.38 | 0.26 - 0.49 | <0.001 |
| Female sex | -0.94 | -1.15 - -0.73 | <0.001 | -0.92 | -1.11 - -0.73 | <0.001 | -0.72 | -0.93 - -0.52 | <0.001 | -0.75 | -0.94 - -0.57 | <0.001 |
| Current smoker | -0.16 | -0.44-0.12 | 0.26 | - | - | - | -0.18 | -0.44-0.09 | 0.19 | - | - | - |
| Body Mass Index (kg/m^2^) | 0.03 | 0.005 - 0.05 | 0.02 | 0.03 | 0.01 - 0.05 | 0.002 | 0.03 | 0.01 - 0.05 | 0.02 | 0.03 | 0.01 - 0.05 | 0.002 |
| History of diabetes | 0.6 | 0.34 - 0.86 | <0.001 | 0.32 | 0.09 - 0.54 | 0.006 | 0.76 | 0.51 - 1.00 | <0.001 | 0.53 | 0.31 - 0.75 | <0.001 |
| History of heart failure | 1.68 | 1.24 - 2.12 | <0.001 | 1.49 | 1.11 - 1.87 | <0.001 | 1.14 | 0.72 - 1.57 | <0.001 | 0.75 | 0.37 - 1.12 | <0.001 |
| Systolic blood pressure (mmHg) | 0.13 | 0.08 - 0.18 | <0.001 | 0.08 | 0.03 - 0.13 | 0.002 | 0.04 | -0.01 - 0.09 | 0.08 | - | - | - |
| eGFR ckd-epi (ml/min/1.73^2^) | -0.28 | -0.35 - -0.21 | <0.001 | - | - | NS | -0.31 | -0.37 - -0.25 | <0.001 | -0.14 | -0.21 - -0.06 | <0.001 |
| Low density lipoprotein cholesterol (mmol/L) | -0.22 | -0.46-0.01 | 0.06 | - | - | - | -0.28 | -0.51-0.06 | 0.01 | - | - | NS |
| *Results presented as unadjusted and adjusted odds ratios with 95% confidence intervals and p-values. Concentrations of cardiac troponin I and T and low-density lipoprotein cholesterol are log_2_-transformed. Age, systolic blood pressure and eGFR are reported per 10-unit increase. Abbreviations: NS - non-significant, eGFR ckd-epi – estimated glomerular filtrations rate using the Chronic Kidney Disease Epidemiology Collaboration equation* | | | | | | | | | | | | |

**Supplemental Table 3**. Predictors of coronary artery luminal stenosis of ≥50% by unadjusted and adjusted logistic regression analyses of the total study population. Only covariates with a *p*-value <0.05 for the association in the univariable model were included in the multivariable model.

|  | **hs-cTnI** | | | | | | **hs-cTnT** | | | | | |
| --- | --- | --- | --- | --- | --- | --- | --- | --- | --- | --- | --- | --- |
|  | **Univariable analyses** | | | **Multivariable analyses** | | | **Univariable analyses** | | | **Multivariable analyses** | | |
|  | **Odds ratio** | **95% Confidence interval** | **p-value** | **Odds ratio** | **95% Confidence interval** | **p-value** | **Odds ratio** | **95% Confidence interval** | **p-value** | **Odds ratio** | **95% Confidence interval** | **p-value** |
| Age (years) | 1.61 | 1.38 - 1.88 | <0.001 | 1.04 | 1.02 - 1.05 | <0.001 | 1.61 | 1.38 - 1.88 | <0.001 | 1.05 | 1.03 - 1.06 | <0.001 |
| Female sex | 0.47 | 0.35 - 0.64 | <0.001 | 0.52 | 0.36 - 0.74 | <0.001 | 0.47 | 0.35 - 0.64 | <0.001 | 0.44 | 0.32 - 0.62 | <0.001 |
| Current smoker | 0.73 | 0.50-1.07 | 0.11 | - | - | - | 0.73 | 0.50-1.07 | 0.11 | - | - | - |
| Body Mass Index (kg/m^2^) | 1.03 | 0.99-1.06 | 0.12 | - | - | - | 1.03 | 0.99-1.06 | 0.12 | - | - | - |
| History of diabetes | 2.02 | 1.38 - 2.96 | <0.001 | 1.63 | 1.08 - 2.45 | 0.02 | 2.02 | 1.38 - 2.96 | <0.001 | 1.77 | 1.19 - 2.65 | 0.005 |
| History of heart failure | 1.13 | 0.61-2.10 | 0.70 | - | - | - | 1.13 | 0.61-2.10 | 0.70 | - | - | - |
| Systolic blood pressure (mmHg) | 1.12 | 1.04 - 1.21 | 0.002 | - | - | NS | 1.12 | 1.04 - 1.21 | 0.002 | - | - | NS |
| eGFR ckd-epi (ml/min/1.73^2^) | 0.78 | 0.70 - 0.86 | <0.001 | - | - | NS | 0.78 | 0.70 - 0.86 | <0.001 | - | - | NS |
| Low density lipoprotein cholesterol (mmol/L) | 0.65 | 0.47 - 0.90 | 0.01 | - | - | NS | 0.65 | 0.47 - 0.90 | 0.01 | - | - | NS |
| hs-cTnI (ng/L) | 1.45 | 1.28-1.64 | <0.001 | 1.20 | 1.05-1.38 | 0.009 | - | - | - | - | - | - |
| hs-cTnT (ng/L) | - | - | - | - | - | - | 1.27 | 1.13-1.41 | <0.001 | - | - | NS |
| *Results presented as unadjusted and adjusted odds ratios with 95% confidence intervals and p-values. Concentrations of cardiac troponin I and T and low-density lipoprotein cholesterol are log_2_-transformed. Age, systolic blood pressure and eGFR are reported per 10-unit increase. Abbreviations: NS - non-significant, eGFR ckd-epi – estimated glomerular filtrations rate using the Chronic Kidney Disease Epidemiology Collaboration equation* | | | | | | | | | | | | |

**Supplemental Table 4**. Predictors of coronary artery luminal stenosis of ≥50% by unadjusted and adjusted logistic regression analyses, stratified by NORRISK2 risk-tertiles. Only covariates with a *p*-value <0.05 for the association in the univariable model were included in the multivariable model.

|  | **Univariable analyses** | | | **Multivariable analyses** | | | **Univariable analyses** | | | **Multivariable analyses** | | | **Univariable analyses** | | | | **Multivariable analyses** | | | |
| --- | --- | --- | --- | --- | --- | --- | --- | --- | --- | --- | --- | --- | --- | --- | --- | --- | --- | --- | --- | --- |
|  | **Low risk tertile** | | | | | | **Intermediate risk tertile** | | | | | | **High risk tertile** | | | | | | | |
|  | **Odds ratio** | **95% Confidence interval** | **p-value** | **Odds ratio** | **95% Confidence interval** | **p-value** | **Odds ratio** | **95% Confidence interval** | **p-value** | **Odds ratio** | **95% Confidence interval** | **p-value** | **Odds ratio** | **95% Confidence interval** | **p-value** | **Odds ratio** | | **95% Confidence interval** | **p-value** |  |
| Age (years) | 1.02 | 0.99-1.05 | 0.18 | - | - | - | 1.01 | 0.97-1.05 | 0.7 | - | - | - | 1.03 | 0.99-1.07 | 0.18 | - | | - | - |  |
| Female sex | 0.55 | 0.33-0.94 | 0.03 | - | - | NS | 0.41 | 0.23-0.72 | 0.002 | 0.38 | 0.21-0.68 | 0.001 | 1.22 | 0.59-2.53 | 0.59 | - | | - | - |  |
| Current smoker | 1.23 | 0.60-2.54 | 0.57 | - | - | - | 0.51 | 0.27-0.98 | 0.04 | - | - | NS | 0.60 | 0.29-1.22 | 0.16 | - | | - | - |  |
| Body Mass Index (kg/m^2^) | 1.05 | 0.99-1.11 | 0.1 | - | - | - | 1.04 | 0.98-1.10 | 0.15 | - | - | - | 1.03 | 0.96-1.11 | 0.44 | - | | - | - |  |
| History of diabetes | 2.72 | 1.32-5.62 | 0.007 | - | - | NS | 1.93 | 1.03-3.61 | 0.04 | 2.07 | 1.09-3.94 | 0.03 | 1.15 | 0.56-2.33 | 0.70 | - | | - | - |  |
| History of heart failure | 0.88 | 0.28-2.77 | 0.83 | - | - | - | 1.56 | 0.56-4.38 | 0.39 | - | - | - | 1.02 | 0.26-3.98 | 0.97 | - | | - | - |  |
| Systolic blood pressure (mmHg) | 1.01 | 1.0-1.03 | 0.17 | - | - | - | 0.99 | 0.97-1.00 | 0.13 | - | - | - | 1.00 | 0.99-1.02 | 0.68 | - | | - | - |  |
| eGFR ckd-epi (ml/min/1.73^2^) | 0.99 | 0.97-1.01 | 0.2 | - | - | - | 0.99 | 0.97-1.01 | 0.27 | - | - | - | 0.99 | 0.97-1.00 | 0.15 | - | | - | - |  |
| Low density lipoprotein cholesterol (mmol/L) | 0.50 | 0.27-0.92 | 0.03 | - | - | NS | 0.50 | 0.28-0.92 | 0.03 | - | - | NS | 0.66 | 0.36-1.20 | 0.17 | - | | - | - |  |
| hs-cTnI (ng/L) | 1.52 | 1.19-1.93 | 0.001 | 1.51 | 1.19-1.92 | 0.001 | 1.23 | 1.01-1.49 | 0.04 | - | - | NS | 1.25 | 0.99-1.58 | 0.06 | - | | - | - |  |
| hs-cTnT (ng/L) | 1.16 | 0.95-1.43 | 0.15 | - | - | - | 1.21 | 0.99-1.48 | 0.06 | - | - | - | 1.00 | 0.80-1.26 | 0.98 | - | | - | - |  |
| *Results presented as unadjusted and adjusted odds ratios with 95% confidence intervals and p-values. Concentrations of cardiac troponin I and T and low-density lipoprotein cholesterol are log2-transformed. Age, systolic blood pressure and eGFR are reported per 10-unit increase. Abbreviations: NS - non-significant, eGFR ckd-epi – estimated glomerular filtrations rate using the Chronic Kidney Disease Epidemiology Collaboration equation* | | | | | | | | | | | | | | | | | | | | |

**Supplemental Table 5**. Sensitivity, specificity, positive predictive values and negative predictive values at the 25^th^, 50^th^ and 75^th^ high-sensitivity cardiac troponin I concentration percentiles in patients with cardiac troponin T concentrations below the Limit of Detection (n=191).

|  |  | **Total population (n=191)** | |
| --- | --- | --- | --- |
| **cTnI cut-off** |  | % (95% CI) | Number of patients with cTn > cut-off (% of group total) |
| **25th percentile (0.8 ng/L)** | Sensitivity | 87 (79-93) | 148 (77) |
|  | Specificity | 32 (23-42) |  |
|  | PPV | 55 (46-63) |  |
|  | NPV | 72 (56-85) |  |
| **50th percentile (1.2 ng/L)** | Sensitivity | 59 (49-69) | 92 (48) |
|  | Specificity | 62 (52-72) |  |
|  | PPV | 60 (49-70) |  |
|  | NPV | 62 (51-71) |  |
| **75th percentile (1.8 ng/L)** | Sensitivity | 33 (24-44) | 47 (25) |
|  | Specificity | 84 (75-90) |  |
|  | PPV | 66 (51-79) |  |
|  | NPV | 57 (48-65) |  |
|  | ROC-AUC | 65 (57-73) |  |

*Abbreviations: cTn – cardiac troponin, PPV – positive predictive value, NPV – negative predictive value, ROC-AUC – area under the receiver operating characteristics curve*

**Supplemental Table 6**. Net Reclassification Improvement, Integrated Discrimination Improvement and Area under the Receiver Operating Characteristics Curve in predicting obstructive coronary artery disease when adding either cardiac troponin I or T to the NORRISK2-score

|  | **hs-cTnI** | | **hs-cTnT** | |
| --- | --- | --- | --- | --- |
|  |  | *95% Confidence interval* |  | *95% Confidence interval* |
| cNRI_event_ | -0.02 | -0.12-0.07 | 0.20 | -0.06-0.27 |
| cNRI_non-event_ | 0.30 | 0.20-0.38 | -0.08 | -0.16-0.04 |
| cNRI | 0.28 | 0.11-0.42 | 0.12 | 0.13-0.25 |
|  |  |  |  |  |
| IDI_event_ | 0.01 | 0.002-0.02 | 0.002 | -0.001-0.008 |
| IDI_non-event_ | 0.01 | 0.003-0.03 | 0.003 | -0.001-0.011 |
| IDI | 0.02 | 0.005-0.05 | 0.004 | -0.002-0.02 |
|  |  |  |  |  |
| ROC-AUC_NORRISK alone_ | 0.65 | 0.61-0.69 | 0.65 | 0.61-0.69 |
| ROC-AUC_NORRISK2 + cTnI_ | 0.67 | 0.63-0.71 | 0.65 | 0.61-0.70 |
| *p*-value for difference | 0.19 | | 0.48 | |

*Abbreviations: hs-cTnI – high-sensitivity cardiac troponin I, hs-cTnT – high-sensitivity cardiac troponin T, cNRI – continuous reclassification improvement, IDI - integrated discrimination improvement, ROC-AUC - Receiver Operating Characteristics - Area Under Curve*

**Supplemental Fig. 1**

Consort flow diagram of patients in the study


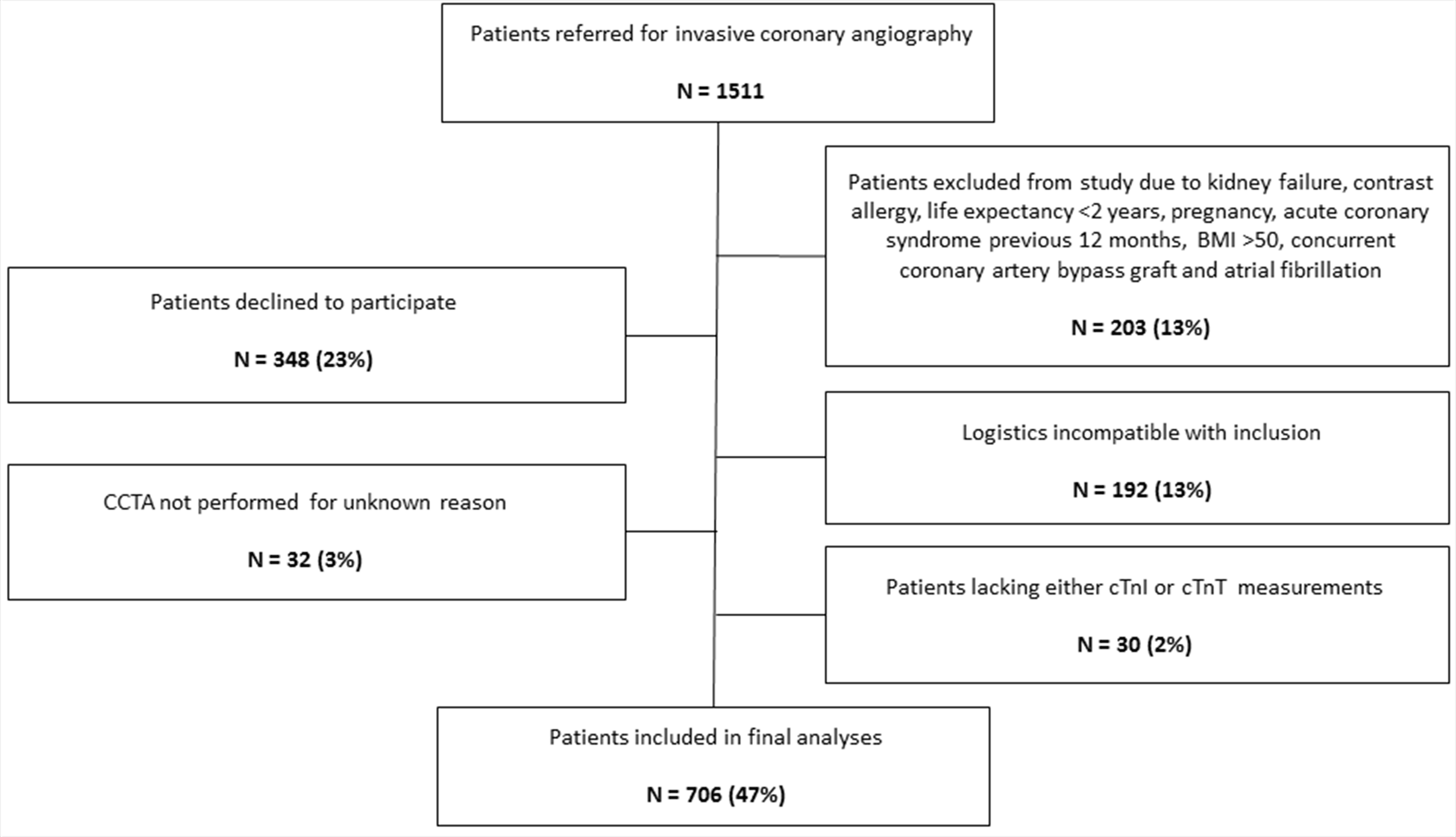


*Abbreviations: CCTA - coronary computed tomography angiography, BMI – body mass index, cTnI – cardiac troponin I, cTnT – cardiac troponin T*
